# Serum zinc concentrations and characteristics of zinc deficiency/marginal deficiency among Japanese subjects

**DOI:** 10.1002/jgf2.377

**Published:** 2020-09-18

**Authors:** Hirohide Yokokawa, Hiroshi Fukuda, Mizue Saita, Taiju Miyagami, Yuichi Takahashi, Teruhiko Hisaoka, Toshio Naito

**Affiliations:** ^1^ Department of General Medicine Juntendo University School of Medicine Tokyo Japan

**Keywords:** aging, deficiency, epidemiology, nutrition, prevention, zinc

## Abstract

**Background:**

Studies that have examined serum zinc deficiency/marginal deficiency in developed countries, including Japan, are still limited. The aim of this study was to assess serum zinc concentrations and associated characteristics among Japanese subjects.

**Methods:**

This cross‐sectional study, conducted from September 2016 to December 2018, included 2056 eligible subjects who participated in a voluntary health checkup. Serum zinc concentration categories were defined as deficiency (<60 μg/dL), marginal deficiency (≥60 to <80 μg/dL), and normal (≥80 μg/dL). Serum zinc concentrations were compared between the first age category (<40 years) and other age categories with Dunnett's method. Trends in P‐values were estimated using the Jonckheere‐Terpstra test for continuous variables.

**Results:**

The proportions of subjects with deficiency and marginal deficiency were 0.4% and 46.0% in men, and 0.6% and 38.4% in women, respectively. The deficiency/marginal deficiency group had significantly lower lipid profiles and nutritional status, and a significantly lower proportion were non–daily drinkers in both genders. Older age was significantly associated with lower serum zinc concentration only in men.

**Conclusions:**

Our findings clarified a high proportion of serum zinc deficiency/marginal deficiency, especially in men, and suggest a possible association between serum zinc levels and nutritional status and alcohol consumption. It may be necessary to manage nutritional status, including zinc intake.

## Graphical abstract

Our cross‐sectional study revealed a high proportion of zinc deficiency/marginal deficiency, with significantly more men experiencing this deficiency than women (46.4% vs 39.0%). Older age showed a negative association with serum zinc levels only in men. The deficiency/marginal deficiency group had significantly lower TC, LDL‐C, TG TP, Alb, and Hb levels, and proportion of non–daily drinker in both genders.

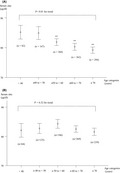

## 1 INTRODUCTION

Zinc is a nutritionally essential trace mineral that is required for the activity of more than 200 enzymes involved in most major metabolic pathways. As such, zinc is necessary for a wide range of biochemical, immunological, and clinical functions.^1^ Mild zinc deficiency may cause impaired taste and smell, reduced immunity, and an increased risk of pneumonia,^2^, ^3^ and severe zinc deficiency may be associated with skin disorders, impaired vision, decreased lymphocyte function, diarrhea, and anorexia.^4^

Zinc deficiency is a major global health issue that affects young children, pregnant women,^5^, ^6^ and the elderly.^7^ Although this issue has received considerable attention in developing countries because of the relationship of zinc concentrations with the risk of malnutrition,^8^ zinc deficiency may be also observed in people in developed countries.^7^, ^9^

However, studies that have examined serum zinc deficiency/marginal deficiency in developed countries, including Japan, are still limited. Therefore, surveys to assess the zinc deficiency are strongly required. The present study aimed to assess serum zinc concentrations and examine the association of serum zinc deficiency/marginal deficiency and other characteristics among Japanese participants who underwent regular health checkups.

## 2 SUBJECTS AND METHODS

This cross‐sectional study screened 5132 Japanese adults who participated in a voluntary health checkup in one Hospital in Tokyo, Japan, from September 2016 to November 2018. Of these participants, 2883 were excluded because of duplicate cases who received health checkup more than 2 times during the study period, and 193 were excluded because of missing data on serum zinc concentrations. As a result, 2056 participants were included in the study. The Ethics Committee of Juntendo University reviewed and approved the research protocol using the retrospective data (No 18‐296), and written comprehensive informed consent was obtained from all participants when they received health checkup.

### 2.1 Variables

Blood samples were collected overnight fast. Serum concentrations of total cholesterol (mg/dL; T‐Cho), high‐density lipoprotein cholesterol (mg/dL; HDL‐C), low‐density lipoprotein cholesterol (mg/dL; LDL‐C), and triglycerides (mg/dL; TG) were also measured. LDL‐C was estimated using the Friedewald equation [(TC) − (HDL‐C) − (TG/5)].^10^ Hemoglobin A1c (HbA1c; National Glycohemoglobin Standardization Program) was determined by high‐performance liquid chromatography using an automated analyzer. As for collection of blood sample, a new specific blood collection tube was used to prevent contamination. Serum zinc concentration (μg/dL) was measured by colorimetric method using a colorimetric reagent kit “ACCURAS AUTO Zn” (SHINO‐TEST Corporation) for determination of zinc in serum. The reagent was applicable to all auto‐analyzer and widely used in Japanese hospital laboratory, without any serum pretreatment. With‐run and between‐run precisions (C.V.) were 0.7‐1.0% and 1.4‐1.9, respectively. The calibration curve was linear up to 500 µg/dL, and the detection limit was 4 µg/dL. A good correlation between the method and those of atomic absorption spectrophotometry was already reported (*r* = .996).^11^ As for the laboratory control, “NIST (SRM3168)” (Shino‐Test Corporation) was used as reference material.

Although serum zinc concentration may not be a reliable indicator of zinc status of an individual, it is widely used and available indicator to assess the risk of zinc deficiency.^9^ Therefore, serum zinc concentration was measured to assess zinc deficiency in the study.

Total protein (g/dL; TP), albumin (g/dL; Alb), hemoglobin (g/dL), and high‐sensitivity C‐reactive protein (mg/dL; hs CRP) levels, and serum uric acid (SUA) (mg/dL) were measured. Estimated glomerular filtration rate (eGFR) was calculated using the Japanese GFR equation: eGFR (mL/min/1.73 m^2^) = 194 × Cr^−1.094^ × age^−0.287^.

Participants were asked to complete a self‐administered questionnaire that addressed healthy lifestyle characteristics based on Breslow's 7 health practices.^12^, ^13^ Healthy lifestyle items in the questionnaire included non–daily alcohol consumption (alcohol consumption 6 or less days per week), non–smoker status, exercise frequency ≥2 times per week, BMI of 18.5‐24.9 kg/m^2^, adequate sleep duration (7 ~ 8 hours), daily breakfast consumption, and no snacking between meals.^12^, ^13^

From the self‐administered questionnaire, we also collected information on medical history of comorbidities, such as diabetes mellitus, dyslipidemia, hyperuricemia/gout, hypertension, cardiovascular disease, and cerebrovascular disease. If participants indicated they had any of these comorbidities, the findings were recorded as being present.

### 2.2 Statistical analysis

Results are presented as mean ± standard deviation (SD) for continuous variables or prevalence (%) for categorical variables. Serum zinc concentration categories were defined following criteria based on the treatment guidelines of zinc deficiency published by The Japanese Society of Clinical Nutrition: deficiency (<60 µg/dL), marginal deficiency (≥60 to <80 µg/dL), and normal (≥80 µg/dL).^14^ For the statistical analysis, “deficiency/marginal deficiency” was combined into a single category of zinc levels <80 μg/dL, because the proportion of subjects with deficiency (<60 μg/dL) was very small (<1.0%).

We used Student's *t* test for continuous variables and the chi‐squared test for categorical variables for comparisons between groups.

In the study, the proportion of participants aged less than 40 years was 7.1%. Therefore, we made a category aged less than 40 years. Among participants aged 40 years or over, we divided into 10 years' category because The Japanese National Health and Nutrition Survey (2017) showed 10 years' age‐stratified zinc intakes.^15^ Serum zinc levels were compared between the first age category (<40 years) and other 10 years' age categories with Dunnett's method. Trends in P‐values were estimated using the Jonckheere‐Terpstra test for serum zinc.

Multivariable regression analysis was performed to estimate the factors associated with serum zinc deficiency/ marginal deficiency among TP (g/dL), Alb (g/dL), Hb (g/dL), T‐Cho (mg/dL), LDL‐C (mg/dL), TG (mg/dL), eGFR (mL/min/1.73 m^2^), and alcohol consumption status, which were significantly different between normal and serum zinc deficiency/marginal deficiency in both genders.

*P* < .05 was considered statistically significant. All statistical analyses were performed using the Statistical Package for Social Sciences, version 22 (SPSS Inc., Chicago, IL, USA).

## 3 RESULTS

**Figure 1:**
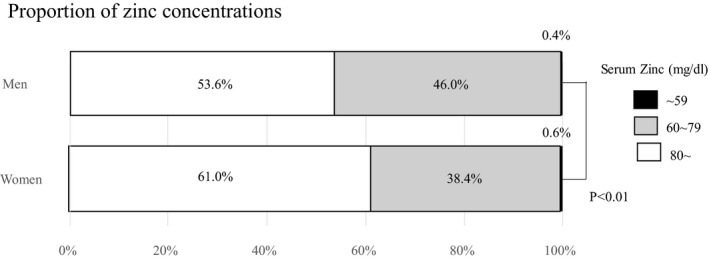
Proportion of zinc concentrations

The proportions of subjects with zinc deficiency (<60 μg/dL) and marginal zinc deficiency (≥60 to <80 μg/dL) were 0.4% and 46.0% in men, and 0.6% and 38.4% in women, respectively (Figure 1). Among 10 participants with zinc deficiency, 5 were men (50.0%). Mean (SD) age, BMI, and serum zinc concentration were 63.8 (14.0) years old, 19.7 (1.3), and 56.7 (3.0) μg/dL, respectively.

Mean age (SD) was 61.9 (13.1) years in the deficiency/marginal deficiency group and 59.3 (12.8) years in the normal group. The proportion of men was 61.3% in the deficiency/mariginal deficiency group and 53.8% in the normal group (Table 1). The deficiency/marginal deficiency group had significantly higher mean Hs CRP measurements, proportion using antihypertensive medication, and a history of heart disease, and significantly lower HbA1c, T‐Cho, LDL‐C, TG, TP, Alb, and eGFR measurements, and proportion of non–daily drinkers.

**Table 1:** Participant characteristics (N = 2056)

	*Mean (SD)* or N (%)				*P*
	Serum zinc concentration (μg/dL)				
	<80 (n = 889)		≥80 (n = 1167)		
Age (years)	*61.9*	*(13.1)*	*59.3*	*(12.8)*	<.01
Gender (men)	545	(61.3%)	628	(53.8%)	<.01
Anthropometric measurements					
Body mass index (kg/m^2^)	*23.3*	*(3.6)*	*23.4*	*(3.6)*	.41
Waist circumference (cm)	*83.1*	*(10.0)*	*83.6*	*(10.0)*	.27
Healthy lifestyle characteristics					
Alcohol consumption (non–daily drinker)	539	(64.6%)	800	(73.6%)	<.01
Smoking behavior (nonsmoker)	731	(87.4%)	937	(86.5%)	.55
Exercise frequency (≥2 times per week)	234	(30.3%)	318	(30.9%)	.80
Body mass index (18.5‐24.9 kg/m^2^)	566	(63.7%)	745	(63.8%)	.96
Adequate sleep duration (yes)	631	(77.8%)	808	(77.0%)	.69
Breakfast (every morning)	665	(79.4%)	884	(81.3%)	.28
Snack between meals (no)	585	(79.3%)	721	(77.0%)	.27
Proportion of participants with 6 or 7 total number of healthy lifestyle items	212	(32.9%)	281	(33.5%)	.82
Hypertensive medication (yes)	200	(22.5%)	210	(18.0%)	.01
Systolic blood pressure (mm Hg)	*124.9*	*(15.9)*	*124.4*	*(15.6)*	.47
Diastolic blood pressure (mm Hg)	*77.1*	*(10.1)*	*77.1*	*(10.2)*	.47
Diabetic medication (yes)	50	(5.6%)	79	(6.8%)	.29
Fasting blood glucose (mg/dL)	*101.5*	*(18.2)*	*101.1*	*(17.4)*	.70
Hemoglobin A1c (%)	*5.8*	*(0.6)*	*5.9*	*(0.6)*	.04
Fasting immunoreactive insulin	*7.7*	*(6.3)*	*8.0*	*(5.4)*	.34
C‐peptide immunoreactivity	1.75	(0.86)	1.71	(0.77)	.03
Dyslipidemia medication (yes)	75	(8.4%)	115	(9.9%)	.27
Total cholesterol (mg/dL)	*201.3*	*(34.3)*	*207.6*	*(34.6)*	<.01
High‐density cholesterol (mg/dL)	*61.8*	*(16.7)*	*61.4*	*(16.3)*	.58
Low‐density cholesterol (mg/dL)	*112.7*	*(28.3)*	*118.4*	*(30.0)*	<.01
Triglyceride (mg/dL)	*108.0*	*(85.0)*	*116.5*	*(84.6)*	.03
Hyperuricemia medication (yes)	32	(3.6)	35	(3.0)	.45
Uric acid (mg/dL)	*5.5*	*(1.3)*	*5.5*	*(1.3)*	.53
Total protein (g/dL)	*7.0*	*(0.4)*	*7.1*	*(0.4)*	<.01
Albumin (g/dL)	*4.2*	*(0.3)*	*4.4*	*(0.3)*	<.01
Hemoglobin (g/dL)	*14.1*	*(1.3)*	*14.3*	*(1.3)*	<.01
High‐sensitivity C‐reactive protein (mg/dL)	*0.14*	*(0.47)*	*0.10*	*(0.26)*	.03
Organ damage/cardiovascular disease					
Cardiovascular disease	52	(5.8%)	43	(3.7%)	.02
Cerebrovascular disease	20	(2.2%)	22	(1.9%)	.56
eGFR (mL/min/1.73 m^2^)	*75.0*	*(16.9)*	*76.6*	*(16.2)*	.03

Abbreviations: eGFR, estimated glomerular filtration rate; N, number; SD, standard deviation.

Table 2 shows gender‐specific characteristics. Although mean age was significantly higher in the deficiency/marginal deficiency group than the normal group in men, no significant differences were observed in women. The deficiency/marginal deficiency group had significantly lower T‐Cho, LDL‐C, TG, TP, Alb, Hb, and proportion of non–daily drinker in both genders.

**Table 2:** Gender‐specific basic characteristics (N = 2056)

	Men (n = 1173)					Women (n = 883)				
	*Mean (SD)* or N (%)					*Mean (SD)* or N (%)				
	Serum zinc concentration (μg/dL)					Serum zinc concentration (μg/dL)				
	<80 (n = 545)		≥80 (n = 628)			<80 (n = 344)		≥80 (n = 539)		
Age (years)	*62.6*	*(12.5)*	*58.2*	*(12.9)*	<.01	*60.7*	*(14.0)*	*60.6*	*(12.5)*	.92
Anthropometric measurements										
Body mass index (kg/m^2^)	*24.3*	*(3.3)*	*24.5*	*(3.2)*	.28	*21.7*	*(3.4)*	*22.2*	*(3.5)*	.06
Waist circumference (cm)	*85.8*	*(9.5)*	*86.7*	*(9.0)*	.10	*78.9*	*(9.3)*	*80.1*	*(9.9)*	.09
Healthy lifestyle characteristics										
Alcohol consumption (non–daily drinker)	285	(55.1%)	366	(63.2%)	<.01	254	(79.9%)	434	(85.4%)	.04
Smoking behavior (nonsmoker)	434	(83.6%)	469	(81.4%)	.34	297	(93.7%)	468	(92.3%)	.45
Exercise frequency (≥2 times per week)	160	(32.9%)	195	(34.8%)	.53	74	(25.9%)	123	(26.2%)	.92
Body mass index (18.5‐24.9 kg/m^2^)	336	(61.7%)	365	(58.1%)	.22	230	(67.1%)	380	(70.5%)	.28
Adequate sleep duration (yes)	402	(79.1%)	447	(79.0%)	.95	229	(75.6%)	361	(74.6%)	.79
Breakfast (every morning)	412	(79.2%)	477	(82.2%)	.21	253	(79.6%)	407	(80.3%)	.80
Snack between meals (no)	369	(79.7%)	384	(75.7%)	.14	216	(78.5%)	337	(78.6%)	.99
Proportion of participants with 6 or 7 total number of healthy lifestyle items	120	(28.9%)	141	(30. %1)	.69	92	(40.2%)	140	(37.7%)	.55
Hypertensive medication (yes)	155	(28.4%)	135	(21.5%)	<.01	45	(13.1%)	75	(13.9%)	.73
Systolic blood pressure (mm Hg)	*126.5*	*(15.2)*	*126.9*	*(14.3)*	.67	*122.4*	*(16.8)*	*121.6*	*(16.5)*	.45
Diastolic blood pressure (mm Hg)	*78.5*	*(9.9)*	*79.2*	*(9.9)*	.25	*75.0*	*(10.2)*	*74.8*	*(10.4)*	.78
Diabetic medication (yes)	44	(8.1%)	63	(10.0%)	.25	6	(1.7%)	16	(3.0%)	.26
Fasting blood glucose (mg/dL)	*105.1*	*(18.6)*	*105.4*	*(19.8)*	.80	*95.6*	*(15.9)*	*96.2*	*(12.3)*	.57
Hemoglobin A1c (%)	*5.9*	*(0.7)*	*5.9*	*(0.7)*	.20	*5.7*	*(0.5)*	*5.8*	*(0.5)*	<.01
Fasting immunoreactive insulin	*8.1*	*(5.5)*	*8.8*	*(5.9)*	.05	*7.2*	*(7.3)*	*7.1*	*(4.5)*	.83
C‐peptide immunoreactivity	*1.9*	*(0.9)*	*1.9*	*(0.8)*	.28	*1.5*	*(0.7)*	*1.5*	*(0.6)*	.45
Dyslipidemia medication (yes)	52	(9.5%)	59	(9.4%)	.93	23	(6.7%)	56	(10.4%)	.06
Total cholesterol (mg/dL)	*195.7*	*(33.0)*	*200.3*	*(34.4)*	.02	*210.0*	*(34.4)*	*216.1*	*(33.0)*	<.01
High‐density cholesterol (mg/dL)	*56.9*	*(15.5)*	*55.4*	*(13.8)*	.10	*69.6*	*(15.6)*	*68.3*	*(16.3)*	.24
Low‐density cholesterol (mg/dL)	*115.1*	*(28.0)*	*119.1*	*(30.4)*	.02	*123.2*	*(29.7)*	*128.5*	*(28.7)*	<.01
Triglyceride (mg/dL)	*121.6*	*(98.2)*	*132.8*	*(100.1)*	.05	*86.6*	*(51.6)*	*97.5*	*(56.2)*	<.01
Hyperuricemia medication (yes)	32	(5.9)	35	(5.6)	.83	0	(0.0)	0	(0.0)	
Uric acid (mg/dL)	*6.0*	*(1.2)*	*6.2*	*(1.2)*	.02	*4.7*	*(1.1)*	*4.8*	*(1.1)*	.18
Total protein (g/dL)	*6.9*	*(0.4)*	*7.1*	*(0.4)*	<.01	*7.0*	*(0.4)*	*7.1*	*(0.3)*	<.01
Albumin (g/dL)	*4.2*	*(0.3)*	*4.4*	*(0.3)*	<.01	*4.2*	*(0.3)*	*4.3*	*(0.2)*	<.01
Hemoglobin (g/dL)	*14.6*	*(1.2)*	*15.1*	*(1.1)*	<.01	*13.2*	*(1.0)*	*13.5*	*(1.0)*	<.01
High‐sensitivity C‐reactive protein (mg/dL)	*0.15*	*(0.48)*	*0.11*	*(0.30)*	.13	*0.12*	*(0.44)*	*0.09*	*(0.21)*	.18
Organ damage										
Cardiovascular disease	42	(7.7%)	31	(4.9%)	.05	10	(2.9%)	12	(2.2%)	.53
Cerebrovascular disease	14	(2.6%)	14	(2.2%)	.70	6	(1.7%)	8	(1.5%)	.76
eGFR (mL/min/1.73 m^2^)	*72.0*	*(14.9)*	*75.7*	*(16.6)*	<.01	*79.8*	*(18.7)*	*77.8*	*(15.8)*	.09

Abbreviations: eGFR, estimated glomerular filtration rate; N, number; SD, standard deviation.

**Figure 2:**
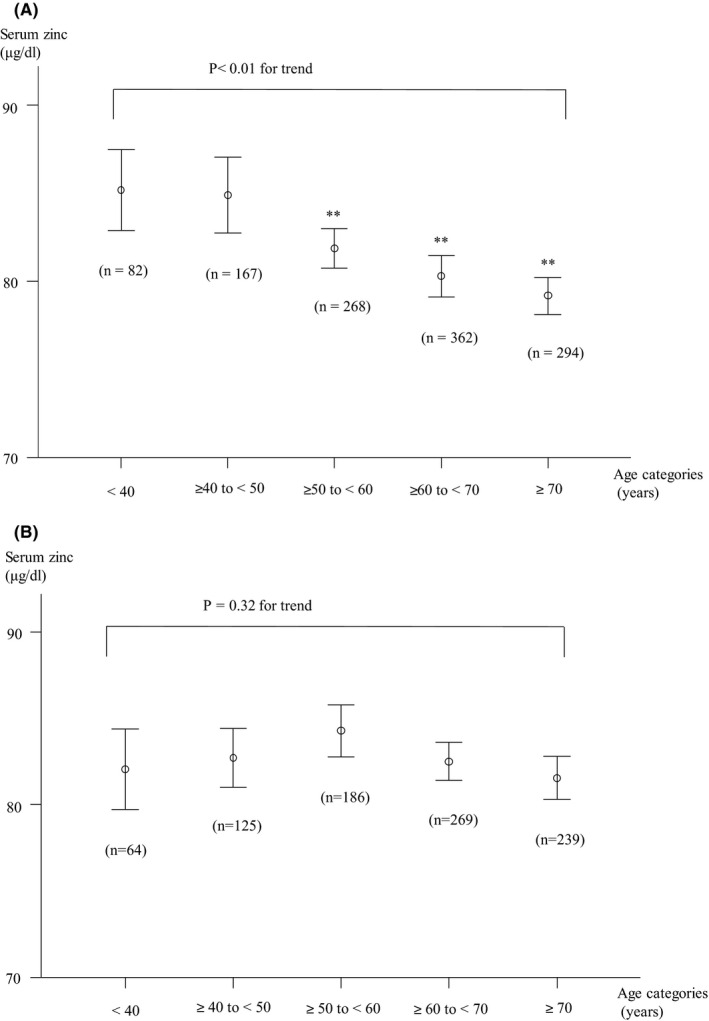
Relationship between age categories and serum zinc concentrations among males (A) and females (B)

Men in the higher age categories (≥50 years) were significantly more likely to have lower serum zinc concentration compared with those <40 years (Figure 2A). However, there was no relationship between age categories and serum zinc concentrations in women (Figure 2B).

Results of multivariable regression analysis showed that non–daily alcohol consumption (OR [odds ratio] = 1.39, 95% CI [confidence interval] = 1.05‐1.84, *P* = .02), Hb (OR = 1.16, 95% CI = 1.02‐1.31, *P* = .03), Alb (OR = 12.49, 95% CI = 6.69‐23.32, *P* < .01), and eGFR (OR = 1.01, 95% CI = 1.00‐1.02, *P* = .04) were significantly associated with serum zinc deficiency/marginal deficiency in men. Among women, non–daily alcohol consumption (OR = 1.62, 95% CI = 1.07‐2.46, *P* = .02), Hb (OR = 1.17, 95% CI = 1.00‐1.36, *P* = .04), Alb (OR = 14.96, 95% CI = 7.09‐31.57, *P* < .01), and TG (OR = 1.01, 95% CI = 1.00‐1.01, *P* = .02) were significantly associated with serum zinc deficiency/marginal deficiency.

## 4 DISCUSSION

Our cross‐sectional study revealed a high proportion of zinc deficiency/marginal deficiency, with significantly more men experiencing this deficiency than women (46.4% vs 39.0%, *P* < .01). Older age showed a negative association with serum zinc levels only in men. The deficiency/marginal deficiency group had significantly lower TC, LDL‐C, TG TP, Alb, and Hb measurements, and proportion of non–daily drinker in both genders. To our knowledge, only a few studies have investigated serum zinc levels and examined the characteristics of serum zinc deficiency/marginal deficiency in a Japanese population. Thus, the present study offers a novel insight on this matter.

Our results clarified that about 40% of those who received voluntary health checkups were likely to experience serum zinc deficiency/marginal deficiency. Marginal deficiency may lead to endocrinological effects such as stunted or delayed puberty in adolescents, dermatitis, reduced appetite, mental lethargy, and taste and smell dysfunction.^16^, ^17^ Also, a Japanese cross‐sectional study reported the favorable association between the serum Zn/Cu ratio and renal function in all subject and glycemic control in patients with type 2 diabetes.^18^ Furthermore, marginal zinc deficiency (<70 μg/dL**)** may cause taste disorders among Japanese adults.^19^ Based on this evidence, The Japanese Society of Clinical Nutrition established treatment guidelines for zinc deficiency in 2018 and recommended a cutoff value of serum zinc concentrations <60 μg/dL to represent deficiency and values ≥60 to <80 μg/dL to represent marginal deficiency.^14^ The Biomarkers of Nutrition for Development Zinc Expert Panel and the International Zinc Nutrition Consultative Group suggest lower cutoffs for serum zinc as follows: females aged ≥10 years, morning/fasting, 70 µg/dL; morning/nonfasting, 66 µg/dL; and afternoon, 59 µg/dL; and males aged ≥10 years, morning/fasting, 74 µg/dL; morning/nonfasting, 70 µg/dL; and afternoon, 61 µg/dL.^20^ However, a few studies have estimated serum zinc concentrations or examined the presence of zinc deficiency/marginal deficiency. An epidemiological study conducted among elderly Japanese people in a rural area showed that the percentage with low serum zinc (2.5th percentile of Americans) was 37.9% in those >60 years, and the age‐adjusted prevalence of low serum zinc was 21.1%.^21^ A survey that examined serum zinc levels among 202 free‐living Japanese women showed that mean serum zinc concentrations were 78 ± 12 μg/dL, and the proportion of those with low and medium serum zinc levels (<82 μg/dL) was 66.3%.^20^ A survey that evaluated serum zinc concentrations in a US population from NHANES 2011‐2014 reported that the mean ± SE serum zinc concentrations in males and females were 84.9 ± 0.8 and 80.6 ± 0.6 μg/dL, respectively.^9^ Although there are variations in serum zinc concentrations, it is possible that 20 to 40% of these people may have serum zinc deficiency/marginal deficiency. Therefore, it is necessary to assess zinc status among free‐living populations as well as subject with taste or smell dysfunction.

Our results showed gender‐ and age‐specific findings associated with serum zinc concentrations. Serum zinc concentrations were significantly lower, and the proportion of serum zinc deficiency/marginal deficiency was significantly higher in men compared with women. A previous study that surveyed serum zinc levels in 1009 Japanese rural inhabitants showed that the proportion of subjects with serum zinc concentrations lower than the cutoff point was significantly higher in men compared with women in those >40 years, whereas no differences were observed among those <40 years.^21^ The Tromsø Study, a cross‐sectional, population‐based survey that evaluated the association between the risk of malnutrition and zinc deficiency among 743 men and 778 women aged 65‐87 years in Norway, reported that zinc deficiency was found in 10.1% of participants, including 13.1% of men and 7.3 % of women.^7^ On the other hand, serum zinc concentrations were significantly higher in men compared with women in a US population (84.9 ± 0.8 and 80.6 ± 0.6 μg/dL, respectively; *P* < .0001).^9^ Therefore, the association between serum zinc deficiency and gender is still controversial. Several possible factors are related to zinc deficiency, including malnutrition, alcohol consumption, and zinc supplementation status.^7^, ^22^, ^23^ Additional studies that include these intermediate factors are needed to determine differences in zinc deficiency between men and women.

Our results showed a negative association between age and serum zinc levels in men, whereas no association was observed in women. Although these results seem paradoxical, a similar result was reported in a US survey.^9^ The previous report showed that serum zinc concentrations were significantly decreased with age in men (87.0 ± 1.6 µg/dL in those aged 19‐30 years vs 81.8 ± 1.8 µg/dL in those aged ≥71 years, *P* = .02), whereas no association with age was seen in women.^9^ On the other hand, the ZENITH study, which surveyed 387 volunteers from France, United Kingdom, and Italy, reported that serum zinc concentrations did not decrease between middle‐age and elderly subjects in either gender (55‐70 years: females, 13.01 ± 1.37 µmol/L, males, 12.98 ± 1.63 µmol/L; >70 years: females, 13.18 ± 2.31 µmol/L, male, 13.23 ± 1.70 µmol/L).^23^ Therefore, age‐specific trends in serum zinc concentrations are also still controversial.

Differences between ages and genders may possibly be explained based on normal zinc intakes. A Japanese survey reported a positive relationship between zinc intake and serum zinc concentrations among elderly subjects (*R* ^2^ = .271).^21^ The Japanese National Health and Nutrition Survey (2017) showed that age‐stratified zinc intakes were 9.1 mg/d among those aged 30‐39 years, 8.8 mg/d among those aged 40‐49 years, 9.2 mg/d among those aged 50‐59 years, 8.7 mg/d among those aged 60‐69 years, and 8.7 mg/d among those aged 70‐79 years among men, whereas respective values were 6.4, 6.8, 7.3, 8.2, and 8.5 mg/d, respectively, among women.^15^ Based on the “Dietary reference intake for Japanese (2015),” established by the Japanese government, intake of 10 mg/d zinc for men and 8 mg/d for women is recommended.^24^ However, the actual intake of zinc was shown to be below the recommended level among middle‐aged and elderly Japanese men, whereas it was sufficient among women of almost all ages.^24^ The Tromsø Study reported that zinc deficiency was found in 12.0% of men and 6.7% of women among those at low risk of malnutrition, whereas 31.0% of men and 12.7% of women had zinc deficiency among those at medium/high risk of malnutrition.^7^ Our data showed that TP, Alb, and lipid profiles were significantly lower in deficiency/marginal zinc deficiency compared to subjects with normal zinc levels in both genders. It is possible that the age‐specific trend in serum zinc concentrations may be related to actual age‐specific nutritional status, although our study could not estimate nutritional status, including zinc intake. Our data may indicate the necessity to manage nutritional status, including zinc intake, among middle‐aged or elderly subjects based on official recommendations.

In the results, SUA was significantly higher in the normal group compared with deficiency/marginal zinc deficiency in men. Generally, serum zinc concentration is likely to be inversely correlated with SUA, and a previous report indicated that dietary zinc intake is inversely associated with hyperuricemia in the United States in both genders, independent of some major confounding factors.^25^ Although our finding seems to be contradicting, there is no association between serum zinc status and SUA in additional logistic regression analysis adjusting lipid profiles, TP, Alb, Hb, alcohol consumption status, and eGFR (*P* = .55). Further analysis focusing on the association between serum zinc status and SUA may be required.

Our study has several limitations. First, selection bias may have occurred, as participants consisted of those who underwent medical checkups in a single medical institution. As such, these participants may be inherently more aware of their health behaviors relative to residents in rural areas. Further analyses that include data from a more diverse cohort are needed. Second, some key data on items such as use of zinc supplements, eating behaviors, nutritional status, clinical symptoms, serum copper concentration, and liver‐related items were not collected. Another prospective studies including the information are needed in future. Although it is necessary to discuss the roles of gender hormones and gut microbiota to assess serum zinc status in aging, it was complicated to investigate in the study.^26^, ^27^ Additional study is needed to discuss the association between serum zinc concentration in aging and these roles.

In conclusion, this cross‐sectional study clarified a high proportion of serum zinc deficiency/marginal deficiency, and a significantly higher prevalence in men compared with women. Older age categories showed a positive linear association with serum zinc levels only in men. The deficiency/marginal deficiency group had significantly lower TC, LDL‐C, TG, TP, Alb, Hb, and proportion of non–daily drinkers in both genders. Collectively, these findings suggest a possible association between serum zinc levels and nutritional status and health behaviors, such as alcohol consumption. It may be necessary to manage nutritional status, including zinc intake.

## CONFLICT OF INTEREST

TN is a supervisor of Nobelpharma Co., Ltd (Tokyo, Japan). Other authors do not have any conflicts of interests.

## ETHICAL APPROVAL

The Ethics Committee of Juntendo University reviewed and approved the research protocol using the retrospective data (No 18‐296), and written comprehensive informed consent was obtained from all participants when they received health checkup.
